# Cysteinyl Leukotrienes in Eosinophil Biology: Functional Roles and Therapeutic Perspectives in Eosinophilic Disorders

**DOI:** 10.3389/fmed.2017.00106

**Published:** 2017-07-18

**Authors:** Glaucia A. Thompson-Souza, Isabella Gropillo, Josiane S. Neves

**Affiliations:** ^1^Institute of Biomedical Sciences, Federal University of Rio de Janeiro, Rio de Janeiro, Brazil

**Keywords:** eosinophils, leukotrienes, granules, cytokine, cysleukotrienes

## Abstract

Cysteinyl leukotrienes (cysLTs), LTC4, and its extracellular metabolites, LTD4 and LTE4, have varied and multiple roles in mediating eosinophilic disorders including host defense against parasitic helminthes and allergic inflammation, especially in the lung and in asthma. CysLTs are known to act through at least 2 receptors termed cysLT1 receptor (CysLT1R) and cysLT2 receptor (CysLT2R). Eosinophils contain a dominant population of cytoplasmic crystalloid granules that store various preformed proteins. Human eosinophils are sources of cysLTs and are known to express the two known cysLTs receptors (CysLTRs). CysLTs can have varied functions on eosinophils, ranging from intracrine regulators of secretion of granule-derived proteins to paracrine/autocrine roles in eosinophil chemotaxis, differentiation, and survival. Lately, it has been recognized the expression of CysLTRs in the membranes of eosinophil granules. Moreover, cysLTs have been shown to evoke secretion from isolated cell-free eosinophil granules operating through their receptors expressed on granule membranes. In this work, we review the functional roles of cysLTs in eosinophil biology. We review cysLTs biosynthesis, their receptors, and argue the intracrine and paracrine/autocrine responses induced by cysLTs in eosinophils and in isolated free extracellular eosinophil granules. We also examine and speculate on the therapeutic relevance of targeting CysLTRs in the treatment of eosinophilic disorders.

## Introduction

**Figure 1:**
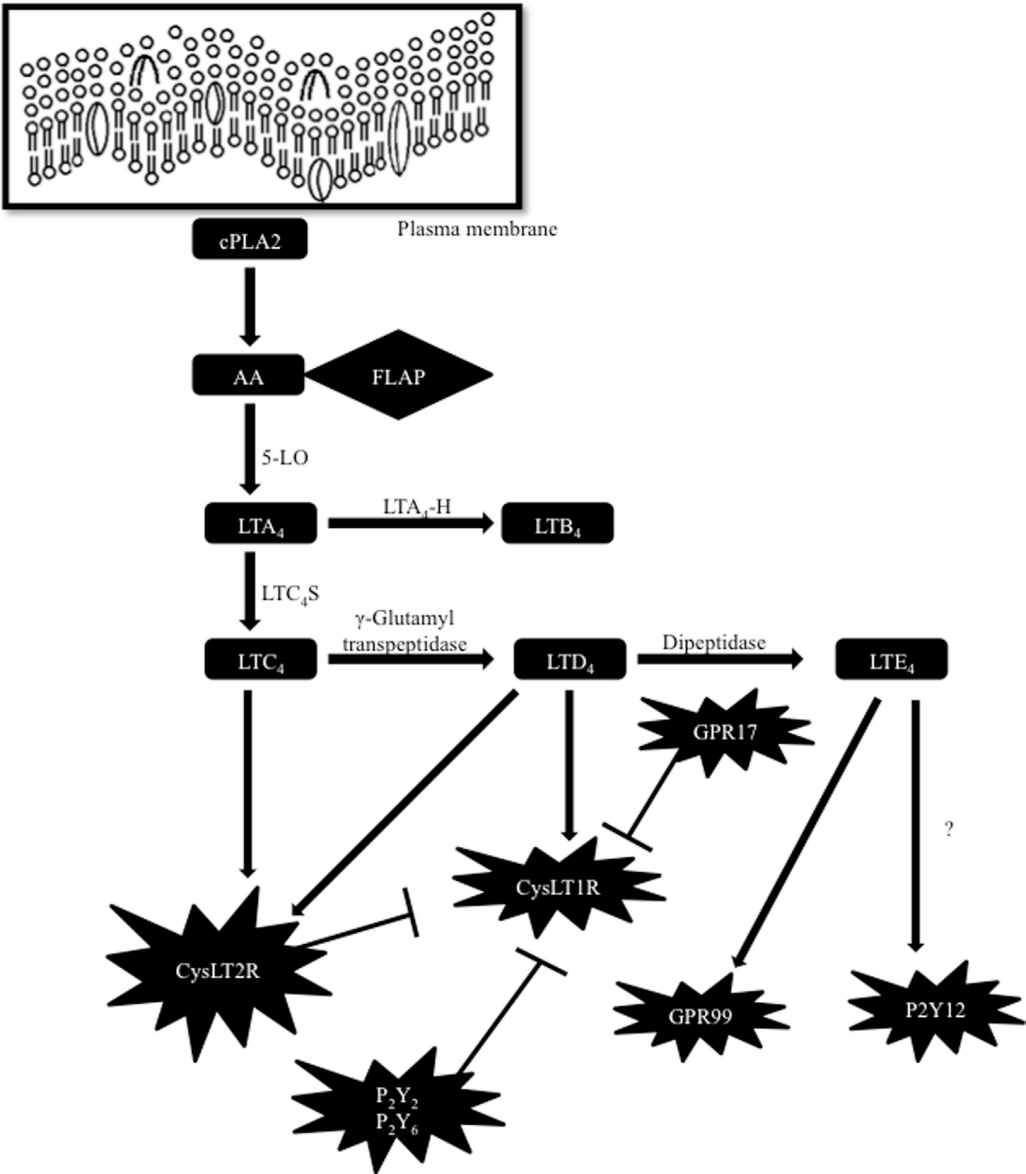
Biosynthetic pathway of cysteinyl leukotrienes (cysLTs) and cross regulation of their receptors. Arachidonic acid (AA) is released from the plasma membrane by a cytosolic phospholipase A2 (cPLA2). To form cysLTs, 5-lipoxygenase-activating protein (FLAP) presents AA to 5-lipoxygenase (5-LO) leading to the formation of leukotriene (LT) A4. LTA4 is rapidly metabolized either to produce LTB4 by the act of LTA4 hydrolase (LTA4-H) or to generate LTC4 by the action of LTC4 synthase (LTC4-S). LTC4 is further enzymatically transformed to LTD4 and LTE4. CysLT2R or GPR17 and PKC-dependent phosphorylation by P2Y receptors inhibit CysLT1R function. P2Y12 receptor (P2Y12R) was primarily identified as a LTE4 ligand, but other studies have suggested that LTE4 does not activate intracellular signaling by acting through P2Y12R. More recently, GPR99 has been suggested as a new receptor sensitive to LTE4.

Lipid mediators such as leukotrienes (LTs) possess multiple cell targets and immunologic functions in different pathological and physiological conditions. LT biosynthesis is initiated throughout the activation of cells, when arachidonic acid (AA) is released from the membrane phospholipids by a calcium-dependent cytosolic phospholipase A2 (1, 2). Free AA is metabolized enzymatically to eicosanoids through at least two major pathways, namely cyclooxygenase (COX) and lipoxygenase (LO) pathways. In the COX pathway, AA is metabolized to prostaglandin H2, which is further metabolized to prostaglandins and thromboxanes by particular prostaglandin and thromboxane synthases. In the LO pathway, AA is metabolized to 8-, 12- and 15-hydroperoxyeicosatetraenoic (HPETE) acids by 12- and 15-LO or to 5-HPETE by 5-LO and 5-lipoxygenase-activating protein (FLAP). FLAP presents AA to 5-LO, which catalyzes the formation of 5-HPETE (1–3). 5-HPETE forms LTA4, which is unstable and rapidly metabolized either to produce LTB4 by the act of LTA4 hydrolase (LTA4-H) or to generate LTC4 by the action of LTC4 synthase (LTC4-S). LTC4 is further enzymatically converted to LTD4 and LTE4 (1, 2) (Figure 1).

Although the biosynthesis of the cysteinyl leukotrienes (cysLTs) mainly occurs in cell-specific compartments, such as the nuclear envelope (4) and specific intracellular organelles called lipid bodies (5) (cytoplasmic organelles rich in lipids that have functions in lipid mediator production), other alternate routes have also been observed in different cells. In eosinophils, basophils, mast cells, and macrophages, LTC4S conjugates LTA4 to reduced glutathione, forming LTC4. Once formed, LTC4 is transported extracellularly *via* the ATP-binding (ABC) proteins and then metabolized to LTD4 and LTE4 by γ-glutamyl transpeptidases and dipeptidases, respectively (2). This process is named cysLTs transcellular biosynthesis and also occurs in other cells, such as endothelial cells, platelets, and even neuronal and glial cells. These cells lack the enzymes to produce LTA4, but they use the LTA4 from the surrounding neutrophils and produce LTC4 [for review, see Ref. (1)].

LTC4, LTD4, and LTE4 are the main ligands for the G-protein-coupled receptors (GPCRs) cysteinyl leukotrienes type 1 (CysLT1R) and type 2 (CysLT2R) receptors. The rank of order is LTD4 > LTC4 > LTE4 by means of their affinity toward CysLT1R (6), whereas CysLT2R binds LTC4 and LTD4 with an affinity one-log less than CysLT1R (binding rank order LTD4 = LTC4 > LTE4) (7). CysLT1R, a high-affinity receptor for LTD4, is expressed in bronchial smooth muscle and substantially in eosinophils, macrophages, and mast cells and is the target of antagonists (montelukast, zafirlukast, and pranlukast) (6). CysLT2R is resistant to montelukast, and is expressed both on cells that also express CysLT1R (e.g., leukocytes) and other tissues.

Different studies have proposed the existence of another cysLT receptor (CysLTR), since several of the cell functions evoked by cysLTs are not well explained by the current knowledge of CysLTRs (8–14). For example, studies performed in mice and humans suggested that LTE4, known as the weakest CysLTRs activator, has biological effects that cannot be elucidated based on its currently accepted affinity to CysLT1R and CysLT2R (11–13). In fact, the purinergic P2Y12 receptor (P2Y12R) has been suggested as a different receptor responsive to LTE4 based on *in vitro* and *in vivo* studies (15, 16). In contrast, different investigations have suggested that cysLTs, including LTE4, do not trigger P2Y12R-mediated intracellular signaling. So, another receptor sensitive to LTE4 has yet to be recognized (17). More recently, a potential new receptor for LTE4 was identified and reported as an oxyglutarate receptor named GPR99 (18) (Figure 1). Current knowledge of CysLT1R and CysLT2R also reveal that CysLTR functions have many non-canonical modulation pathways. Now it is known that CysLT1R can be regulated by indirect or direct physical interactions with other GPCRs. For instance, protein kinase C activation by the purinergic P2Y2 and P2Y6 receptors can induce phosphorylation and desensitization of CysLT1R, when these receptors are coexpressed in cell lines, without causing CysLT1R internalization (19). Moreover, in human mast cells, CysLT1R and CysLT2R heterodimerize (20), limiting the levels of membrane expression of CysLT1R as well as its functional signaling capacity. GPR17, a GPCR homologous to CysLT1R and CysLT2R, was first characterized as a dual-specific receptor for cysLTs and uracil nucleotides (21). Nevertheless, in further studies, it was revealed that GPR17 operates as a negative regulator of CysLTR1 activation induced by LTD4 and distinctly reduces binding of LTD4 in cells that express both classes of receptors (22) (Figure 1). Thus, more investigations are needed in order to better understand the many unpredictable responses obtained in the studies with cysLTs. Potentially, many other direct or indirect interactions, that are still unknown, may exist among CysLTRs and other GPCRs.

Currently, eosinophils are defined as multifunctional cells that have long been related to allergy and host parasite responses. They are immunomodulatory cells that contribute both in innate and adaptive immune responses *via* the selective secretion of different cytokines and other mediators. CysLTs and CysLTRs have significant roles in allergic conditions and are valuable pharmacological therapeutic targets for the control of asthma and other eosinophilic diseases [for review, see Ref. (23)]. Human eosinophils are main producers of cysLTs and express both CysLT1R and CysLT2R on their cell plasma membranes (2, 24). Among other GPCRs capable of potentially responding to cysLTs or interacting with CysLTRs, it is now recognized that eosinophils express the P2Y2R, P2Y6R [for review, see Ref. (25)], P2Y12R (26), and the GPR99 (27). However, the functional roles of these receptors as regulators of CysLTRs in eosinophils are still not known. The expression of GPR17 in eosinophils has not been identified so far.

Mature human eosinophils are easily differentiated by the abundant presence of secretory granules termed crystalloid, secondary, or even specific granules (28). Eosinophils are also characterized by a vesicular system and lipid bodies, in which various lipid mediators are synthesized. Within eosinophils, synthesis of LTC4 (but not LTD4 or LTE4) occurs at perinuclear membranes and in cytoplasmic lipid bodies (24, 29). Eosinophil crystalloid granules present a unique morphology with a central crystalline core compartment surrounded by a matrix, which is delimited by a trilaminar membrane. These granules express different receptors in their wrapping trilaminar membrane and store a large number of preformed proteins such as cytotoxic cationic proteins and many cytokines and chemokines. Human eosinophils synthesize and store cationic proteins such as eosinophil peroxidase, eosinophil cationic protein (ECP), eosinophil granule major basic protein, and eosinophil-derived neurotoxin (EDN). They also biosynthesize, store, and selectively secrete growth factors, enzymes, chemokines (such as eotaxin and RANTES), and over more than three dozen cytokines in response to different stimuli (28, 30–35). Piecemeal degranulation (PMD), a process by which granule contents are selectively mobilized into vesicles that arise from the granules and fuse with the plasma membrane to extracellularly release their cargo, is the major mechanism of intact eosinophil granule protein secretion (32, 36). A different mechanism of human eosinophil “degranulation” is known as cytolysis, which involves damage of eosinophil cell membrane integrity, release, and deposition of cell-free membrane-bound crystalloid granules to the extracellular microenvironment. Even though PMD is recognized as the main mechanism operating during eosinophil protein secretion, cytolysis has been considered the main mechanism underlying the release and tissue deposition of intact, membrane-bound free eosinophil granules observed in different eosinophilic diseases. Exocytosis, whereby the entire granules fuse with the plasma membrane releasing their content extracellularly, has been considered a more unusual mechanism of eosinophil secretion and it is not usually observed *in vivo* (35, 37).

## Functional Roles of cysLTs in Eosinophil Biology

Over the last years, a number of mediators (cytokines, chemokines, growth factors, alarmins, and lipid mediators) involved in the regulation of eosinophil recruitment, degranulation, survival, and other functions have been identified. A rising bulk of data has revealed essential roles of cysLTs in regulating different eosinophil functions.

**Figure 2:**
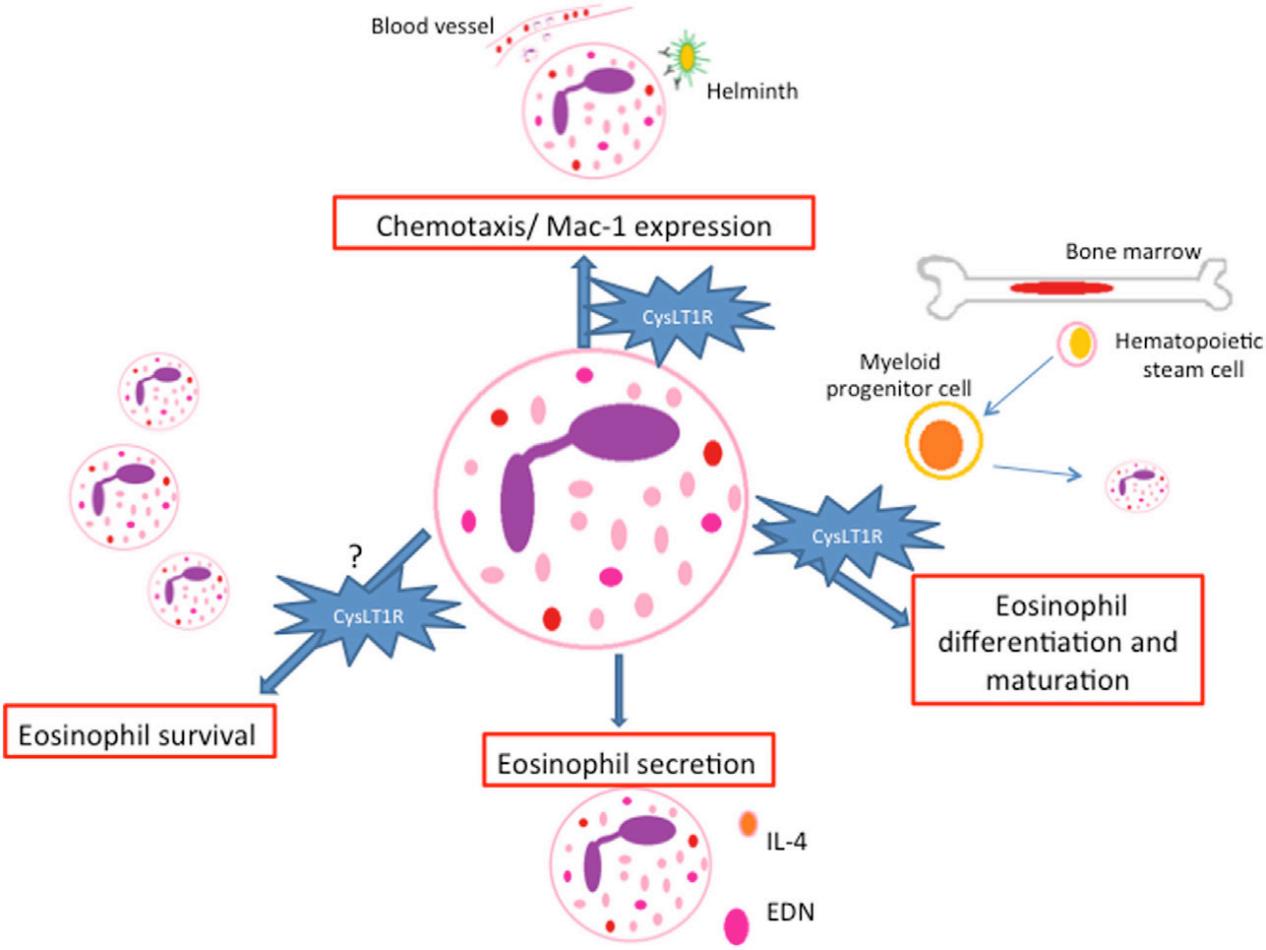
Paracrine/autocrine responses evoked by cysteinyl leukotrienes (cysLTs) in eosinophils. CysLTs mediate different eosinophil functions such as chemotaxis, eosinophil differentiation and maturation, survival and protein secretion, most of them *via* the CysLT1R. EDN, eosinophil-derived neurotoxin.

It has been reported that cysLTs display eosinophilotactic activity *in vitro via* CysLT1R (38–40) (Figure 2). These studies revealed that LTD4 may act as a potent and selective eosinophilotactic factor at physiological concentrations (38) and in directly increasing Mac-1 expression in a mechanism dependent on CysLT1R (39). Further data also show that LTD4 induced eosinophil transendothelial migration across human umbilical vein endothelial cells in a Pranlukast (a CysLT1R antagonist) dependent manner (40). *In vivo*, involvement of cysLTs in eosinophil influx was firstly demonstrated in guinea pigs in the 90s (41), and further in humans (42). Subsequently, these finding were reinforced by the effects of CysLT1R antagonists in inhibiting eosinophil recruitment in airway allergic inflammation (43, 44). Recently, roles for LTC4 in mediating eosinophil trafficking from lungs to paratracheal lymph nodes in experimental allergic asthma were described (45).

Regarding eosinophil secretory functions, published data show that LTD4 induced eosinophil ROS generation and EDN release. Pranlukast significantly inhibited EDN release, although the inhibitory effect on ROS generation was partial (40). In a different study, cysLTs induced the release of IL-4 from human cord blood progenitor derived-eosinophils in a dose- and time-dependent manner (46) (Figure 2).

CysLT1R antagonists also appear to play a role in limiting IL-5-responsive eosinophilopoeisis, since cysLTs and IL-5 act together at several stages of eosinophil differentiation and maturation during upper airway allergic inflammation (47). In addition, cysLTs also appear to enhance the *in vitro* survival of human eosinophils by activation of CysLT1R (48, 49) (Figure 2). Though it has been demonstrated that eosinophils isolated from asthmatic patients can have their apoptosis postponed by cysLTs, controversial data suggest that the cysLTs, despite raising intracellular calcium, are unable to prolong survival of eosinophils isolated from normal individuals or mildly atopic patients (50).

**Figure 3:**
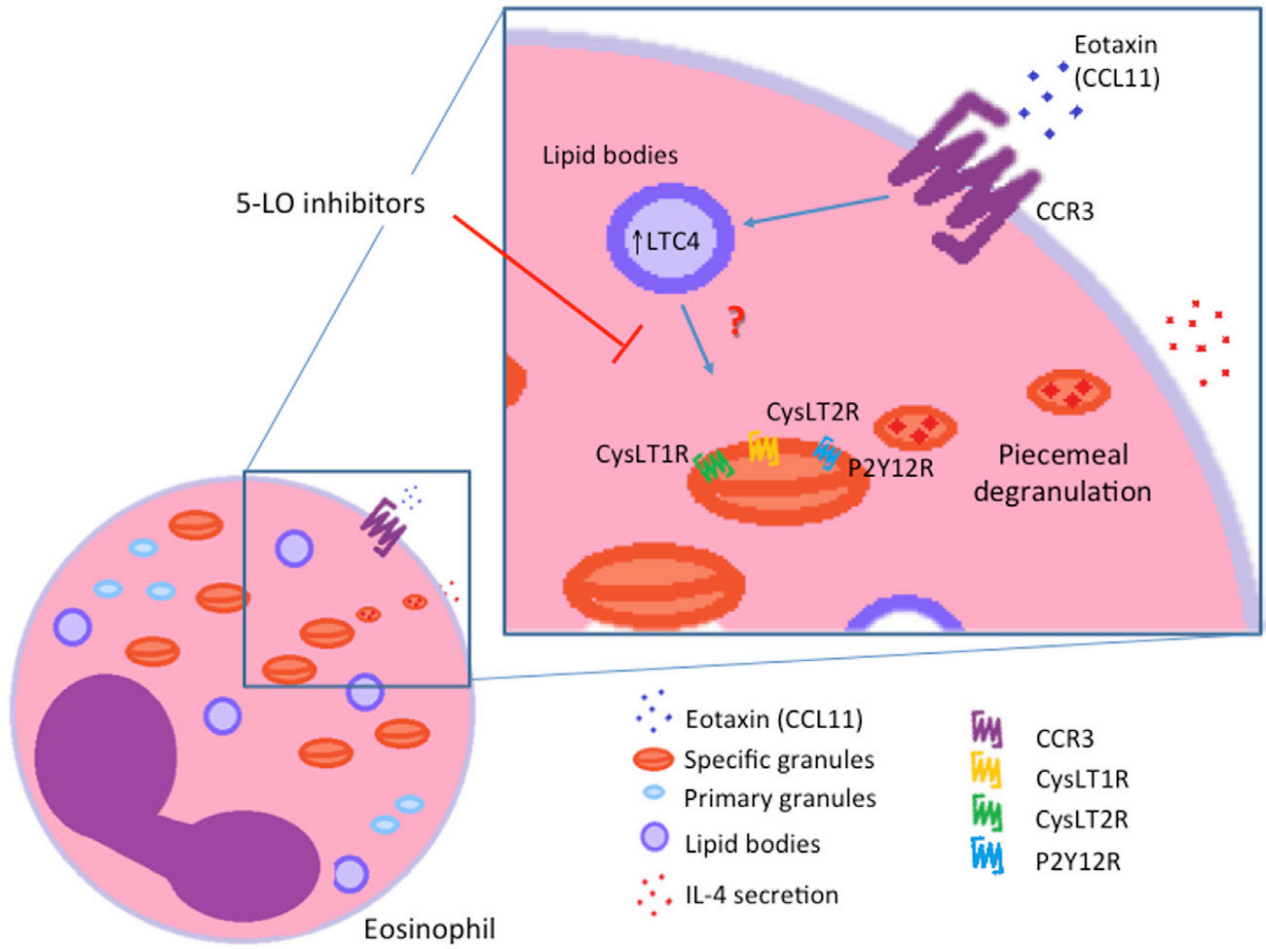
Intracrine actions of cysteinyl leukotriene (cysLTs). IL-4 release induced by eotaxin (CCL11) is dependent on the intracrine action of lipid body-generated LTC4. Inhibitors of 5-lypoxigenase (5-LO) blocked intracellular LTC4 production and consequently IL-4 release from eosinophils. CysLT1R, cysLT1 receptor; CysLT2R, cysLT2 receptor; P2Y12R, purinergic P2Y12 receptor.

It is well established that eosinophils are major sources of cysLTs (24). Beyond their functions as paracrine mediators, cysLTs are now also known to exhibit autocrine and likewise intracrine effects. Lee and colleagues provided evidence for the involvement of an autocrine cysLT pathway that is involved in eosinophil survival in response to GM-CSF (48). Interestingly, it is also described that LTC4 can be synthesized in different intracellular compartments (nuclear membrane or lipid bodies) and may function as intracrine regulators of selective granule protein secretion (5, 51, 52). In 2002, Bandeira-Melo and colleagues (51) demonstrated that eotaxin (CCL11) stimulates human eosinophil to secrete IL-4 by PMD in a lipid body-generated LTC4-dependent mechanism. The authors also showed that 5-LO blockers inhibited the IL-4 secretion. In this way, the intracellular-formed LTC4 would function as an intracrine signaling molecule, mediating CCR3-induced IL-4 release (Figure 3). Exogenous LTC4 and LTD4 at low concentrations induced IL-4 release (but not RANTES) only after membrane permeabilization. Inhibitors of the CysLT1R and CysLT2R did not block LTC4-elicited IL-4 release suggesting that LTC4, *via* an intracellular CysLTR distinct from CysLT1R and CysLT2R, may also function as an intracrine mediator capable to trigger cytokine secretion *via* PMD. Another work that explored leukotrienes as possible intracrine mediators of eosinophils’ PMD is a study published by Tedla and colleagues (52). The authors showed that the cross-linking of immobilized antibodies and CD9 and leukocyte immunoglobulin-like receptor 7 (LIR7) stimulates human eosinophil to secrete IL-12 (but not IL-4) by PMD and to generate LTC4 at perinuclear regions (52). However, pretreatment of eosinophils with two different inhibitors of 5-LO did not inhibit this selective release of IL-12 (52). These findings indicate that CD9- or LIR7-induced selective IL-12 release is not dependent on 5-LO action. Based on these studies, it has been suggested that lipid body-generated LTC4 may relate less to paracrine mediator formation and more to intracrine signaling functions. However, more studies are still needed in order to better clarify this point.

Although eosinophils express different GPCRs capable of potentially responding to cysLTs or interacting with CysLTRs, little is known about the intracellular distribution of these receptors in eosinophils. Recently, the expression of cysLT-responsive receptors has been recognized on the delimiting trilaminar membrane of intracellular crystalloid eosinophil granules. These receptors function mediating cysLT-evoked secretion from cell-free eosinophil granules protein content (26, 53). However, whether these receptors have roles when these granules are in the cytoplasmic microenvironment it is not known.

## Functional Roles of cysLTs in Cell-Free Functional Extracellular Eosinophil Granules

Intracrine roles for cysLTs have been reported; however, the possible mechanisms that can elucidate the intracellular activities of cysLTs remain unknown (51, 54, 55). Recently, our group demonstrated that free eosinophil granules express CysLT1R and CysLT2R and the P2Y12R on their membranes (26). In addition, formerly, it was demonstrated that eosinophil granules are enriched sites of various cytokine and chemokine receptors (31, 53, 56); and that these granules, upon extrusion from eosinophils, responded to CCL11 and IFN-γ, through their granule membrane-expressed receptors. The activation of the receptors triggered signaling pathways within granules that promote protein secretion (53, 57). Isolated free eosinophil granules stimulated with cysLTs secrete ECP, but not chemokines or cytokines. CysLT1R or P2Y12R blockage inhibited the eosinophil granule ECP secretion. The capacity of both CysLT1R and the P2Y12R antagonists to similarly inhibit ECP secretion elicited by cysLTs, including LTE4, might suggest functional heterodimerization or cross regulation of CysLT1R with other GPCRs. However, so far this is not clearly defined. Remarkably, the dose response to the three cysLTs differed. LTC4 and LTE4 induced ECP release only at subnanomolar concentrations, which was coherent with the GPCRs’ typical high-dose inhibition. Interestingly, LTD4 induced ECP secretion at low and high concentrations. At intermediate concentrations, LTD4 was unable to promote granule ECP secretion. As mentioned earlier, whether dimerization or cross regulation of GPCRs are involved in this response remains to be elucidated. However, considering the variable results of studies with cysLTs, what is certain is that there are pieces to this puzzle that are still missing. These studies highlight the ability of cysLTs to evoke isolated free granule secretory functions. Moreover, for granules functioning as cytoplasmic organelles, these studies reveal new mechanisms by which LTC4 and extracellularly formed LTD4 and LTE4 (after cellular uptake) may operate as intracrine signaling molecules capable to induce eosinophil granule protein secretion. Nevertheless, this is no evidence that the CysLTRs or the P2Y12R present on the trilaminar granule membranes participate in the intracrine cysLTs’ actions reported earlier (51) (Figure 3). So far, more studies are needed in order to elucidate whether the eosinophil granule membrane-expressed receptors mediate intracrine actions of cysLTs.

## Targeting CysLTRs in the Treatment of Eosinophilic Disorders: Concluding Remarks and Questions for the Future

Among eosinophilic disorders, the CysLT1R blockers (zafirlukast, montelukast, and pranlukast) are mainly used in the management of some chronic respiratory diseases, particularly allergic rhinitis and bronchial asthma. In fact, in the management of asthma, the current clinical data are in favor of their use as an add-on or alternative therapy to inhaled corticosteroids (58, 59). Clinical trials evaluating zafirlukast, montelukast, and pranlukast have shown a decrease of eosinophil count in blood and airways of asthmatic patients (60, 61). However, other studies (62, 63) suggest that the development of dual CysLT1/2R antagonists might bring additional advantages to the asthma treatment over the current used CysLT1R blockers. In fact, patients with chronic persistent asthma presented superior improvement in lung function when treated with a cysLT synthesis inhibitor compared to a CysLT1R antagonist (62). However, recently, a clinical study with a dual CysLT1/2R blocker, the compound ONO-6950, in non-smoking subjects with mild allergic asthma, showed no additional benefits of this therapeutic strategy to the treatment of asthma (64).

Besides the two classic receptors for cysLTs (CysLT1R and CysLT2R), there remain important questions regarding the potential clinical implications of novel receptors for cysLTs or the cross regulation of CysLT1R. Current knowledge is the only beginning to understand the molecular pharmacology of the receptors sensitive to CysLTRs, their capacity to cross regulate or to signalize as dimers. So far, physiological and pharmacological reports have shown a great complexity and functional variation of the cysLT system. Important questions remain about the regulation of the CysLT1R by other GPCRs and its potential clinical relevance. For instance, considering that CysLT2R and GPR17 negatively regulate CysLT1R function, is it likely that functional diversification in each receptor could impact clinical relevance to CysLT1R antagonists? Other intriguing analysis can be performed regarding the purinergic receptors versus their sensitivity to CysLT1R antagonists, and their capacity to induce desensitization of the CysLT1R. Is it thinkable that the capacity of CysLT1R antagonists to inhibit these presumed negative regulators of the CysLT1R could (i) impair the benefits of their use, (ii) reduce the potential therapeutic benefit for some patients, and (iii) explain some of the heterogeneity of response to these agents? Moreover, considering the increased sensitivity of asthmatic patients to LTE4, and the fact that LTE4 has a long half-life and is abundantly found in asthmatic patients, would GPR99 blockers be in the horizon as an innovation for asthma treatment? To end, are cell-free secretory extracellular eosinophil granules new therapeutic targets beyond intact eosinophils for all these antagonists? So far, these and other questions remain unanswered.

## Author Contributions

GT-S, IG, and JN conducted a review of the literature. GT-S and JN wrote the manuscript.

## Conflict of Interest Statement

The authors declare that the research was conducted in the absence of any commercial or financial relationships that could be construed as a potential conflict of interest.
